# Current trends in virtual electrophysiology use for risk stratification and treatment of ventricular arrhythmias

**DOI:** 10.3389/fcvm.2025.1709175

**Published:** 2025-11-20

**Authors:** Christos-Konstantinos Antoniou, Konstantinos Karampinos, Dimitrios Tsiachris, Athanasios Kordalis, Petros Arsenos, Ioannis Doundoulakis, Polychronis Dilaveris, Nikias Milaras, Skevos Sideris, Ourania Kariki, Alexandros Kasiakogias, Charalambos Vlachopoulos, Konstantinos Toutouzas, Konstantinos Tsioufis, Konstantinos Gatzoulis

**Affiliations:** 1 First Department of Cardiology, “Hippokration” Hospital, National and Kapodistrian University of Athens, Athens, Greece; 2 Third Department of Cardiology, “Sotiria” Thoracic Diseases General Hospital, National and Kapodistrian University of Athens, Athens, Greece; 3 State Department of Cardiology, “Hippokration” General Hospital of Athens, Athens, Greece; 4 Department of Cardiology, “Onassis” Cardiac Surgery Center, Athens, Greece

**Keywords:** cardiac simulation, virtual electrophysiology, arrhythmic risk stratification, ventricular arrhythmia ablation, artificial intelligence

## Abstract

Currently efforts are being undertaken to establish and bring into clinical practice the field of virtual cardiac electrophysiology. The basic premise lies in acquiring an accurate whole-heart model based both on anatomy and electrophysiological properties of every myocardial voxel. Subsequently, one option is to perform a virtual electrophysiology study, with no constraints regarding site and number of extrasystoles in order to assess arrhythmogenic potential of the ventricle (ventricular arrhythmia risk prediction). The alternative, in cases with documented ventricular arrhythmia, would be to fine-tune the model into being able to simulate the clinical arrhythmia and then assess its mechanism, establishing vulnerable sites and thus ablation targets in order to guide the subsequent interventional procedure (virtual arrhythmia ablation targeting). Once clinical evidence supports vEP value in terms of accuracy and safety, it could be expected that even induced, nonclinical, arrhythmias could be targeted. Finally, advances in the field of computational power and artificial intelligence, including radiomics, along with stereotactic arrhythmia radioablation could render the future of arrhythmia management and treatment virtually unrecognizable in the not-so-distant future. The present mini review will attempt to familiarize clinicians with the tenets and current state of vEP, especially in the current phase where larger prospective clinical studies are required for further advancement, as well as offer a glimpse at potential future directions of this approach.

## Introduction

Following the advent of percutaneous revascularization as a milestone in combatting ischemic heart disease, sudden cardiac death prevention and primary treatment of malignant ventricular arrhythmias are at the forefront of contemporary research in cardiovascular medicine, not least due to the significant associated mortality and morbidity burden (1). In the majority of cases, sudden cardiac death is tachyarrhythmic in nature—thus effective prevention is inherently linked to accurate assessment of ventricular tachy-arrhythmogenic potential (2). On the other hand, primary treatment of clinical ventricular tachyarrhythmias though ablation is a major and often laborious undertaking, requiring precise guidance as to the targets where energy (in whichever form) should be applied in order to suppress arrhythmogenesis (3–5). Obviously, one could further add the potential for ablation of potential/induced (in silico or *in vivo*) arrhythmias, however clinical data are sparser and less encouraging regarding this approach (6, 7) -at least in the context of “conventional” (*in vivo*) electrophysiology and arrhythmia mapping/ablation (itself an ever-evolving field).

In contrast to the above, virtual—in silico—electrophysiology (vEP), both diagnostic and therapeutic, could be considered to include all methods aiming to yield accurate whole-heart simulations, which can then be used to either study general arrhythmogenesis mechanisms or specifically assess arrhythmogenic potential in a given individual and thus determine sudden death risk (ventricular arrhythmia risk prediction—VARP) (8–10). Moreover, those same approaches can be employed in order to model a clinical arrhythmia in a specific patient, determine its mechanism, and guide ablative treatment to critical regions, leading to arrhythmia suppression—virtual heart arrhythmia ablation targeting (VAAT) (11, 12).

Consequently, the necessary components of vEP can be broadly categorized as follows:

- Collection of data sufficient to yield an accurate whole heart simulation. Such data include both anatomical (e.g., dense scar and grey zone location, intermediate fibrosis presence) and functional (e.g., conduction velocity and refractory period) components. Furthermore, data can be either generic—i.e., allocating experimentally known electrophysiological properties to each cardiac tissue category or personalized; that is, importing anatomical data, as well as conduction velocity and substrate, from invasive electroanatomical mapping (13–15).
- Assembling the model and performing virtual electroanatomical study on it. This is the most taxing part of the process, computation-wise, and explains why most simulations only extend to 2–3 s after arrhythmia triggering (16). Notably, implantable cardioverter-defibrillators (ICDs) are never programmed to detect, much less treat, an arrhythmia after so short an interval from initiation. However, it stands to reason that, should the model suggest formation of an enduring arrhythmia mechanism, it should negate the need for protracted simulation.

In cases of vEP being used as a guide to malignant ventricular tachyarrhythmia treatment, modifications of the above are necessary:

- The sinus rhythm-based model (with either generic or personalized data) must be able to accurately simulate the clinical arrhythmia(s) (11, 17). The aim is to successfully determine the vulnerable parts of the circuit, or, more generally, the critical myocardial areas where energy application and ablation will lead to arrhythmia termination. A notable advantage of accurate whole-heart simulations is guidance for ablation of nonclinical induced arrhythmias, as well as assessing the effect of lesion administration on arrhythmogenetic potential—i.e., lesions themselves may lead to formation of new circuits which require further ablation.

The approaches which have been developed to achieve these goals, all following the aforementioned principles, are presented below.

## General approaches

One fundamental decision regarding whole heart simulation concerns use of the mono- or bidomain cardiac electrophysiology model (18, 19). The bidomain-based approach considers intracellular and extracellular spaces as distinct and interacting ionic current pools, thus offering higher accuracy. Moreover, the bidomain approach is able to include and assess the differential effects of arrhythmogenesis or scar in these 2 pools, e.g., gradual intracellular calcium overload that may ultimately lead to conduction block or a triggered activity based depolarization that could lead to propagation wave break and arrhythmia degeneration, or the presence of areas acting as current sinks that could destabilize potential isthmuses, respectively. Even the effects of mechanosensitive ion channel activation during heart failure exacerbation could, in theory, be simulated. However, this uncompromising approach is extremely computationally taxing and has not been pursued in all clinically oriented studies, at least given the currently available computational power. The monodomain approach has not been shown to be significantly inferior to the bidomain one, and entails considering a single pool of ions, currents, and voltage changes. Usually (8, 20, 21), experimentally measured concentrations of ionic channels for Na^+^, K^+^, and Ca^++^ are assigned to each voxel (i.e., fundamental volume element), with modifications based on the voxel belonging to normal tissue, gray zone, or dense scar. As a principle, scar is considered electrically inert whereas, at the gray zone peak sodium current, peak L-type calcium current, IKr, and IKs are set to 38%, 31%, 30% and 20% of the values usually assigned to normal myocardium per the Ten Tusscher model, respectively. This modifies the action potential morphology, rendering similar to that experimentally observed. Furthermore (21), changes in connexin-43 concentrations and localization (usually 90% reduction in transverse connectivity) can also be introduced to the model, affecting conduction anisotropy. Consequently, conduction velocities, wavefront propagation direction, and refractory periods can be inferred.

Voxels themselves, as well as their size, originate from the imaging method employed in acquiring the anatomical component of the simulation (20, 22–25). Both cardiac magnetic resonance (CMR) and multidetector computed tomography (MDCT) have been used, each with distinct advantages. More specifically, CMR is superior in determining tissue properties (normal myocardium, grey zone, and dense scar), based on presence and type of fibrosis. Indeed, it has long been recognized that CMR may provide valuable data for potential circuit localization (26, 27). VT isthmuses are always located in conducting channels inside the scar area, and most such channels contain isthmuses (28). However, threshold dependence should be considered. i.e.,: changing LGE threshold will alter conduction channel metrics and model prediction/simulation accuracy—underscoring the need for robust quantification (29).

On the other hand, MDCT has higher spatial resolution, more consistency in findings (30), better delineates anatomical boundaries and lipid infiltration (often crucial in late onset arrhythmogenesis), and can (at least partially) assess fibrosis presence based on tissue thickness. In fact, a study showed that all ventricular tachycardia channels are located in MDCT-detected isthmuses, whereas half of isthmuses contain such channels (25). Following image acquisition, myocardial fiber orientation can be introduced by means of a rule-based method, assuming rotation from +60° in the endocardium to −60° in the epicardium (31).

Indeed, image integration into three-dimensional electroanatomical mapping (3D-EAM) systems offers valuable information regarding scar characterization. Studies (32) have reported improved outcomes (both acute—non-inducibility) and at follow up. Notably, a recent multicenter study evaluating two VT ablation workflows, an “imaging-aided” one—incorporating preprocedural imaging to facilitate mapping by providing the anatomical component of 3D-EAM—and an “imaging-guided” one—whereby ablation targets were determined by preprocedural imaging, and no additional invasive mapping was performed prior to ablation—found no difference in VT-free survival between the two groups. The “image-guided” approach was, however, significantly faster. It is important to note that the study lacked a comparison group using a non-imaging-based ablation strategy (33).

Obviously, information regarding substrate properties (i.e., fibrosis presence) and conduction orientation and velocity can also be obtained through invasive 3D-EAM (10). Recent introduction of omnipolar potential-based mapping (34, 35) is expected to further improve accuracy and spatial analysis of 3D-EAM. However, at least when compared to classical bi- and unipolar mapping, models based on assigned EP properties have been found on par with those including input from 3D-EAM, obviating the need for invasive assessment (36). Of note, the cardiac conduction system is usually not modeled in current clinical vEP studies due to difficulties in rendering accurate fiber course—although its electrophysiological properties are known (10).

Once a complete model has been developed, virtual programmed stimulation may be performed from as many sites as desired; usually, however, 19 sites (corresponding to the 17 segments of the left ventricle per ASE, plus right ventricular apex and base) are used (8, 9, 37). Extrastimuli are usually limited to 3 (i.e., up to an S4). Herein lies a distinct advantage of vEP since arrhythmia induction is more likely if the programmed stimulation is conducted from an adjacent site. Furthermore, mechanical effects from catheter pressure/overzealous manipulation are by definition absent. However, as stated previously, only a short post-stimulation time can be simulated, not longer than a few seconds, and indeed, a study found that should simulation be extended to 10seconds, there was better match with clinical arrhythmias, with 41% of “sustained” arrhythmias at 3 s terminating before the 10 s mark (16).

Regarding vEP as a means to guide ablative treatment of malignant ventricular arrhythmias, it should be underscored once more that its application is currently limited to clinical, not inducible, arrhythmias. Ventricular tachycardia ablation is a taxing procedure, associated with risks—overall complication rate exceeding 13% and mortality rates ranging from 5% in older cohorts to 1.8% in newer patient series (38–41). Both extensive ablation and/or repeated arrhythmia induction termination can, in the context of severely impaired contractility, lead to myocardial stunning and cardiogenic shock—which is why pre-emptive mechanical circulatory support is often required. Thus, removing the need for extensive mapping and prolonged/extensive ablation is desired, and may prove pivotal in rendering ablative treatment a more appealing option.

A 12 lead ECG is required in order to fine-tune model properties until the clinical arrhythmia is simulated (42). Consequently, the mechanism can be visualized and target points highlighted on the anatomical shell. These data can then be transferred to a mapping system and guide energy application to the same sites, not requiring tedious mapping and repeated arrhythmia induction/termination. Crucially, this approach can not only guide ablation of all patient-specific arrhythmias but can also be extended to guide ablation of all additional *potential* arrhythmias in such a patient. Moreover, lesions can be conceived as tissue rendered inactive and introduction of actual, as opposed to proposed, lesions into the model and reiteration of VARP can verify that the patient is no longer inducible. Should this not be the case, or a new arrhythmia occur in the modified substrate, further lesion sets are proposed until VARP yields no further arrhythmogenesis (11).

Despite the above approach being based on sound physiological principles, it should be highlighted that no universally accepted protocols or standards exist for vEP model construction. Moreover, dynamic factors, such as autonomic tone, electrolyte changes, active ischemia, and drug effects are often not fully modelled, affecting the ability to predict arrhythmogenesis, particularly ventricular arrhythmogenesis, under stress or variable conditions which dynamically affect tissue electrophysiological properties (conduction velocity and refractoriness). In any case, such conditions are limitations for invasive electrophysiological studies as well. In theory, the bidomain model could account for electrolyte disorders (yet the computing power necessary for its application is currently unavailable) and data acquisition from myocardial tissue under stress/ischemia/medication could assist in simulating such conditions (but are currently unavailable as well).

## (Pre)clinical studies

Overall, clinical evidence for vEP effectiveness is limited, with head-to-head prospective comparisons with currently established risk stratification and ventricular arrhythmia ablation approaches absent. Rather, most studies focus on either a retrospective, proof of concept, approach, or on small, case series-like, prospective cohorts.

As early as 2009 (43) efforts had begun to acquire an anatomically accurate whole heart model, initially focusing on ex vivo cardiac preparations from small animals. A few years later (44), vEP in the context of VARP started to be performed on *in vivo* animal models of post-myocardial infarction tachycardia, with encouraging results, compared to invasive programmed stimulation—6/7 swine inducible on programmed stimulation were also inducible on VEPR, while all suggested reentrant circuits were similar to the actual ones, if only with reverse propagation in some cases.

Important publications concerning potential for the translational potential of the above came in 2016 with the group of Arevalo et al. publishing on the use of VARP in post-infarction patients throughout the ejection fraction (EF) spectrum (8, 9). In those patients with an EF < 35% (*n* = 41) inducibility upon VARP was significantly associated with the primary endpoint of appropriate ICD activation or sudden cardiac death, with a fourfold hazard ratio. Notably, performance of all other potential risk stratifiers, including EF as well as newer parameters, such as scar volume, grey zone volume, and left ventricular mass was disheartening overall (hazard ratios close to 1 in all cases). Moreover, VARP significantly outperformed invasive programmed stimulation in a subset of 32 patients who had had both conducted. Concerning those with an EF ≥ 35% (9), a much smaller cohort of 4 patients (mean EF 44%), retrospectively assessed, yielded encouraging results for the VARP approach, given that the 1 patient with known monomorphic ventricular tachycardia was inducible on VARP and, additionally, the proposed circuit coincided with the actual circuit delineated during the ablation procedure. All other patients were not inducible on VARP and had no clinical arrhythmic events—obviously, larger studies in non-bearers of ICDs will significantly benefit from the presence of an implantable loop recorder.

VAAT was initially tested in a 2018 study (11), which included all steps, from preclinical, animal, application to retrospective, and then prospective, human patient involvement. Interestingly, proposed lesion volume was smaller than actual volume in the prospective studies (both animal and human). Although in the prospective cohort the ablated arrhythmia was induced, rather than clinical, ablation was performed *without any invasive mapping,* based on VAAT alone, and was associated with postprocedural noninducibility in all cases. Another small study found that, as stated previously, VAAT significantly decreases ablation sites, up to *fifteenfold*, as well as ablated volume by a factor of 2 (45). Novel techniques allow for VAAT utilization to consider lipid infiltration of the myocardium, a known proarrhythmic phenomenon (12). Notably, in this study a CT (rather than CMR)-based model was constructed and, in a cohort of 29 ischemic patients having already been submitted to ablation for monomorphic ventricular tachycardia, proposed lesions not only were associated with significantly less ablated myocardial volume (almost a quarter of what had been deemed necessary in the ablation procedure) but also predicted successful ablation sites, more so in cases of apical infarcts. Even more encouragingly, in 6 cases that necessitated a redo procedure, the proposed sets of ablation sites corresponded to the successful ablation sites at the redo procedure.

Recently, integration of artificial intelligence (AI) approaches for machine learning, extending from support vector machine to convoluted neural networks, has been attempted (37). Indeed, when compared to inducibility upon VARP, AI-augmented vEP had and accuracy of almost 90% in predicting arrhythmogenesis when provided with data from computational models—thus significantly enhancing efficacy and reducing time needed for VARP.

Despite the encouraging results mentioned, currently no universally accepted protocols or standards exist for vEP model construction, particularly clinical validation, or reporting, strongly limiting reproducibility and broad clinical adoption.

## Current and future perspectives

Most workflows described (except those dependent on invasively procured anatomy and EP values) depend on image-derived tissue characterization (core vs. border zone). Radiomics, extracting tissue features invisible to the naked eye and supported by artificial intelligence are currently poised for entering clinical practice for cardiomyopathies (46–49). Radiomics can both quantify scar heterogeneity/texture beyond simple visual cues and absolute thresholds and subsequently inform model parameters (such as regional conductivity) to refine risk and target predictions (50). Thus, this approach will replace or augment fixed intensity cut-offs with texture-based heterogeneity indices to set tissue classes and conduction parameters more objectively. Leading to a more accurate model and consequently simulation.

However, although radiomics can capture subtle patterns in cardiac MRI, a major limitation lies in that these features are not yet clearly linked to underlying tissue characteristics such as fibrosis, scar, or inflammation—analogous to the reported black-box phenomenon of artificial intelligence. As a result, their biological meaning and mechanistic integration into simulations or corridor mapping remain uncertain. Future work should focus on validating radiomic signatures against histology and other imaging modalities, so they can evolve from statistical predictors into physiologically grounded tools for risk stratification and digital-twin modelling. Nevertheless, a radiomics-informed digital twin for VARP and VAAT remains a potential path forward.

Most studies have so far focused on ischemic cardiomyopathy, likely due to the relative stability of substrate between acute ischemic episodes, which renders ventricular arrhythmogenesis more easily predictable. It thus stands to reason that in the future attempts will be made to expand VARP and VAAT into other cardiomyopathies as well (51).

A major limitation of both vEP components (VARP and VAAT) currently lies in the available computational power, which often necessitates hours-long modelling. Quantum computing, despite a lull after an exaggerated initial hype, continues to wield significant advantages over classical computing due to the potential for parallel calculations, excelling in modelling and optimization problems, which are obviously indispensable for VARP and VAAT (52). Moreover, the more accurate bidomain model, allowing for simulation of additional phenomena, as well as longer simulated time periods post arrhythmia induction will be feasible. In any case, shortening the necessary time to produce a whole-heart model capable of simulating arrhythmogenesis and arrhythmia mechanism and critical sites will lead to a valid and viable alternative to currently espoused risk stratification approaches. Obviously, further incorporation of AI, along with advances in AI itself, will further reduce necessary computing time and increase accuracy of both VARP and VAAT. Obviously, as with human training, if data inserted in the training cohort (acquired by CMR, MDCT, invasive EP for modelling and actual successful ablation sites for VAAT) are inaccurate or irrelevant, results will be poor, as with recent developments in the PROFID project (53).

Another future perspective for VARP lies in determining whether a cardiac resynchronization therapy (CRT)—defibrillator or CRT—pacemaker device is indicated in a given patient, a dilemma which often appears during clinical practice (54). It is known that CRT *per se* exerts antiarrhythmic effects through multiple mechanisms (55, 56) and, moreover, whole-heart simulation may allow for assessing the effect of pacing through different/multiple sites (i.e., CRT) on global ventricular contractility (57). Subsequently, the effect of a specific chosen pacing configuration on ventricular arrhythmogenesis may be evaluated.

An initially fringe arrhythmic risk stratification approach that has been steadily gaining traction and acceptance among the electrophysiological community is the tiered two-step approach with presence of noninvasive risk factors leading to invasive programmed ventricular stimulation. This approach has been repeatedly validated in post-infarction ischemic heart disease with EF > 40% (58, 59) and is currently being evaluated in dilated cardiomyopathy across the whole EF spectrum (60). Obviously, substituting VARP for invasive programmed stimulation would render this approach potentially more accurate and certainly more patient-friendly. Certain non-invasive risk factors, already shown to be associated with inducibility upon invasive programmed ventricular stimulation (61), as well as with patient outcomes (62), such as presence of late potentials on signal-averaged electrocardiogram (denoting presence of slow conduction areas in the myocardium) (63), could act as indicators for the initiation of VARP and, if necessary, VAAT.

Should large clinical studies and trials establish vEP usefulness as a tool for risk stratification and arrhythmia prediction, the next logical step would be to actually act on its findings, in a strategy similar to preventive ventricular tachycardia ablation (6) (i.e., targeting an induced rather than clinical arrhythmia), instead of implanting a cardioverter-defibrillator. Pursuing this approach will be favored by the ability to target *all* ventricular tachycardias inducible for a given substrate, as well as by the simplification and shortening of the ablation procedure itself, leading to reduced complications and to (currently unproven) higher short- and long-term success rates.

Whole heart modeling, including components of conduction system, could allow for vEP expansion to also include prediction of bradyarrhythmia occurrence, invaluable in the assessment of syncope (64). However, initially, simpler, more straightforward cases may be analyzed, e.g., atrioventricular block and need for permanent pacemaker implantation post-transcatheter aortic valve replacement, or even supraventricular arrhythmia occurrence and mechanism. Admittedly, no relevant data are currently available, save a study having used the vEP approach to visualize and guide ablation of the cavotricuspid isthmus for atrial flutter treatment.

Moreover, given the recently published ESC position statement for stereotactic arrhythmia radioablation (STAR) (65) is it reasonable to assume that it could be coupled with vEP, augmented with quantum computing, leading to SCD risk assessment and ventricular arrhythmia ablation (clinical and/or in silico inducible) transforming into something akin to a regularly repeatable outpatient procedure in the foreseeable future.

Finally, it is noteworthy that different editions of the software necessary to run whole heart simulations based on imaging and electrophysiological data (such as CARP, life^x^, and Chaste) are currently available online as freeware (66–68); their respective sites being: https://opencarp.org/, https://lifex.gitlab.io/, and https://chaste.github.io/. Others are available in modular format, e.g.,: https://github.com/vildenst/3D-heart-models. Thus, research groups focusing on the field of vEP could design studies and clinical trials without the daunting task of developing ex nihilo models, benefitting from the work of previous researchers.

However, one should not expect to witness the full implementation of the complete spectrum of vEP in electrophysiology labs in the near future. Promising results do exist, but small cohorts, retrospective design, computational demands, and incomplete physiology simulation are the main barriers before routine clinical use of virtual electrophysiology in the treatment of ventricular arrhythmias.

## Conclusions

**Figure 1:**
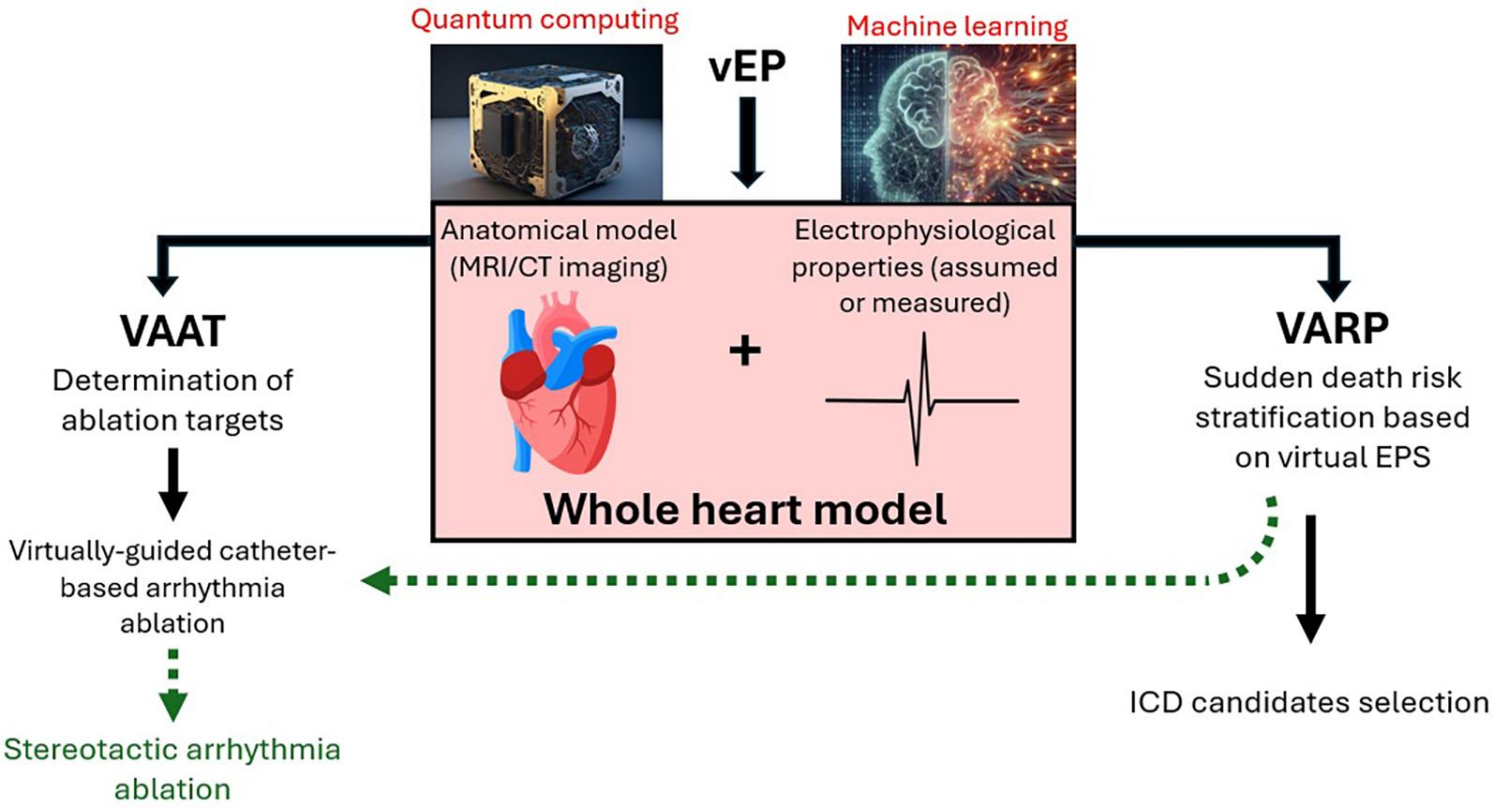
Schematic representation of vEP. Possible augmentations (red font) and potential future directions (green arrows) are also included. Images reproduced from: “Ai Generated Quantum Computer” by TheDigitalArtist, licensed under Content License; “Ai Generated Mathematics” by SamOcean, licensed under Content License; “Heart Anatomy” by BlenderTimer, licensed under Content License.

In the near future, virtual electrophysiology and its components (VARP and VAAT) are poised to revolutionize our approach to sudden cardiac death risk stratification and ventricular arrhythmia primary prevention and treatment (Figure 1). Noninvasively extracting data concerning arrhythmogenic potential of a ventricle, as well as guiding ablation procedure into consuming a fraction of the time and applying a fraction of the energy it would previously have been necessary will make these approaches potentially more accurate, and certainly patient-friendly and safer. There are undeniable and evident current limitations of the vEP approach in terms of simulation accuracy (model construction and dynamic condition simulation), computational power and clinical validation. However, these should not be considered irremediable disadvantages but rather diseases of a field in its infancy, with the potential for significant improvement. Large, prospective clinical studies are sine qua non to firmly establish the validity of the vEP approach, however, should AI-based and quantum computing-based augmentations become available, one might envision, in the not-too-distant future, sudden cardiac death becoming a rare, if tragic, occurrence.
